# Single-cell and bulk transcriptomic characterization of the osteosarcoma microenvironment reveals lineage-specific programs and inflammatory differences associated with metastasis

**DOI:** 10.3389/fgene.2026.1927885

**Published:** 2026-10-16

**Authors:** Zhiwen Zhou, Zhijun Li

**Affiliations:** 1 Department of Orthopedics, Zhongda Hospital, Southeast University, Nanjing, Jiangsu Province, China; 2 Department of Joint Surgery, The Fifth Affiliated Hospital of Southern Medical University, Guangzhou, Guangdong Province, China

**Keywords:** osteosarcoma, single-cell RNA sequencing, tumor microenvironment, osteoblasts, metastasis, bulk transcriptomics, ligand-receptor interactions, RUNX2

## Abstract

**Background:**

Osteosarcoma is a heterogeneous bone malignancy in which malignant, immune, stromal, and bone-lineage cells form a complex microenvironment. Clinical activity of immune-checkpoint blockade has remained limited in advanced osteosarcoma, motivating cell-type-resolved investigation of the tumor microenvironment while avoiding mechanistic overinterpretation of cross-sectional transcriptomic data.

**Methods:**

Single-cell RNA-sequencing data from primary osteosarcoma were obtained from GEO (GSE162454; six patients) and analyzed with Scanpy using quality filtering, normalization, highly variable gene selection, principal component analysis, neighborhood graph construction, UMAP, Leiden clustering, and marker-based cell-type annotation. Seven transcriptional programs and ligand-receptor communication scores were evaluated. An independent bulk osteosarcoma cohort with metastatic status and overall survival annotation (GSE21257; n = 53) was used for differential expression, cross-platform comparison, and survival analysis. RT-qPCR was performed in primary human osteoblasts exposed to MG-63 osteosarcoma-conditioned medium.

**Results:**

After quality control, 44,189 cells with 25,473 detected genes were retained. Leiden clustering resolved 23 clusters annotated into 11 cell types; myeloid cells (34.0%) and osteoblasts (16.6%) were the largest populations. Osteoblasts showed the highest osteogenic program scores, whereas myeloid cells showed the highest inflammatory and immune-evasion scores. Reclustering of osteoblasts, osteocytes, and mesenchymal stromal cells resolved seven stromal subclusters, but pairwise co-expression between representative osteogenic and inflammatory genes was generally weak. Ligand-receptor analysis highlighted MIF-CD74, SPP1-CD44, and SPP1-ITGAV as the strongest scored interactions. In GSE21257, 333 genes differed between metastatic and non-metastatic tumors at FDR<0.05; the inflammatory module was lower in metastatic tumors, whereas the osteogenic module was not significantly different. The single-cell osteoblast effect showed only modest concordance with the bulk metastatic effect (Spearman rho = 0.254), and a 20-gene osteoblast signature did not stratify overall survival (log-rank p = 0.968). *In vitro*, osteosarcoma-conditioned medium reduced RUNX2 and SP7 and increased SOST and RANKL expression in primary osteoblasts.

**Conclusion:**

Integrated single-cell and bulk transcriptomic analyses identify lineage-specific osteogenic, inflammatory, and communication programs in osteosarcoma but also demonstrate limited cross-platform generalizability of osteoblast-centered signatures. The RT-qPCR experiment supports an osteoblast transcriptional response to soluble factors from MG-63 cells, but it does not establish a specific immunosuppressive mechanism or prognostic biomarker. These findings should therefore be considered hypothesis-generating and require independent single-cell, spatial, and functional validation.

## Introduction

Osteosarcoma (OS) is the most common primary malignant bone tumor, occurring most frequently in adolescents with a second incidence peak in older adults (Mirabello et al., 2009). Population-based and cooperative-group studies report survival around 60% for younger or non-metastatic patients and markedly poorer outcomes for metastatic disease (Mirabello et al., 2009; Bielack et al., 2002). Contemporary reviews likewise emphasize that survival gains have plateaued despite modern multimodal therapy, particularly in metastatic or recurrent disease (Isakoff et al., 2015; Luetke et al., 2014).

The limited improvement in outcomes for metastatic or recurrent osteosarcoma has motivated increasing interest in the tumor microenvironment and immune-based therapeutic strategies. A prospective phase 2 study of pembrolizumab in advanced osteosarcoma reported no clinically significant antitumor activity (Boye et al., 2021), consistent with the low response rate observed in the osteosarcoma cohort of SARC028 (Tawbi et al., 2017). Osteosarcoma contains diverse malignant, immune, stromal, and bone-lineage populations whose relative abundance and transcriptional states may influence disease behavior (Liu et al., 2021; Kansara et al., 2014; Corre et al., 2020; Zhu et al., 2022). Rather than assuming a uniformly immunosuppressive architecture, a major analytical challenge is to define which cellular programs are reproducibly associated with specific lineages and whether those programs generalize across independent datasets.

The bone and osteosarcoma microenvironment is a dynamic system in which osteoblasts, osteoclasts, mesenchymal stromal cells (MSCs), immune populations, and malignant cells participate in reciprocal signaling and remodeling (Liu et al., 2021; Kansara et al., 2014; Corre et al., 2020; Zhu et al., 2022; Lorenzo et al., 2008). Osteoblast differentiation is governed by core transcription factors including RUNX2 and SP7 (Osterix) (Komori, 2006; Nakashima et al., 2002). Mature osteocytes further regulate bone remodeling through sclerostin (SOST)-mediated Wnt antagonism and RANKL-dependent osteoclastogenesis (Poole et al., 2005; Xiong et al., 2011; Baron and Kneissel, 2013). These established osteobiological pathways provide a framework for interpreting lineage-specific transcriptional programs in osteosarcoma without assuming that expression changes alone demonstrate causal immune regulation.

Prior work suggests that osteosarcoma-associated inflammatory signals, bone-remodeling pathways, and Wnt-related programs may influence interactions among malignant cells, osteoblast-lineage cells, myeloid populations, and other stromal components (Liu et al., 2021; Kansara et al., 2014; Corre et al., 2020; Zhu et al., 2022; Baron and Kneissel, 2013). However, transcriptomic co-expression and ligand-receptor scoring are inferential measurements and cannot by themselves establish the direction or causality of these interactions. Accordingly, an analysis that combines cell-type-resolved transcriptional profiling with an external bulk cohort can help distinguish robust lineage-associated signals from signatures that do not generalize across platforms or clinical groups.

Single-cell RNA sequencing (scRNA-seq) enables high-resolution characterization of cellular heterogeneity and lineage-specific transcriptional states within complex tumors (Tanay and Regev, 2017; Luecken and Theis, 2019). It can also support exploratory reconstruction of putative cell-cell communication networks from ligand-receptor expression patterns (Armingol et al., 2021). Nevertheless, single-cell findings require careful validation because clustering parameters, patient-of-origin effects, cohort composition, and platform differences can influence apparent cell states (Luecken and Theis, 2019). Independent cohorts and orthogonal datasets are therefore important for evaluating the reproducibility and clinical relevance of candidate signatures.

Here, we analyzed primary osteosarcoma scRNA-seq data from six patients (GEO: GSE162454) to define cellular composition, lineage-specific transcriptional programs, stromal heterogeneity, and expression-based ligand-receptor interactions. The source study generated one primary osteosarcoma specimen from each of six treatment-naive patients, with specimens collected before neoadjuvant chemotherapy (Liu et al., 2021). Individual-level clinical variables were not used for stratified analyses because the public GEO metadata were incomplete for some characteristics. We then used an independent bulk osteosarcoma cohort (GSE21257) to test metastatic differential expression, cross-platform concordance, and survival relevance of an osteoblast-centered signature. Finally, we assessed four osteoblast-related transcripts by RT-qPCR after exposing primary human osteoblasts to MG-63 osteosarcoma-conditioned medium. The study was designed as an exploratory, hypothesis-generating analysis, with particular attention to the limits of cross-platform and mechanistic interpretation.

## Methods

### Single-cell RNA-seq data acquisition and preprocessing

Single-cell RNA-sequencing (scRNA-seq) data of primary osteosarcoma (GSE162454; 10x Genomics; platform GPL24676) were obtained from the NCBI Gene Expression Omnibus (GEO) (Liu et al., 2021). Count matrices from six patients (GSM4952363-GSM4952365, GSM5155198-GSM5155200) were read with scanpy (Wolf et al., 2018). Cells with fewer than 200 or more than 7,000 detected genes and cells with more than 20% mitochondrial reads were excluded; genes detected in fewer than 3 cells were removed. Library sizes were normalized to 10,000 counts per cell and log1p-transformed.

### Dimensionality reduction, clustering, and cell-type annotation

The 2,000 most highly variable genes were used for principal component analysis (50 components), and a k-nearest-neighbor graph was constructed with 15 neighbors and 30 principal components. UMAP (McInnes et al., 2018) embedding and Leiden (Traag et al., 2019) clustering (resolution 0.8, igraph flavor) were applied to the graph. For each Leiden cluster, marker genes were identified by Wilcoxon rank-sum tests of cluster versus rest; cell-type identity was assigned by weighted overlap of the top 50 markers against a curated lineage panel (T cells, NK cells, B cells, plasma cells, myeloid cells, endothelial cells, fibroblasts, osteoblasts, osteoclasts, osteocytes, MSC, tumor/OS).

### Program scores and cell-cell communication

Seven transcriptional programs (osteogenic, inflammatory, immune evasion, hypoxia, EMT, bone remodeling, stemness) were scored per cell by the mean expression of curated gene sets. Ligand-receptor communication scores were computed as the product of the mean ligand expression in the sender cell type and the mean receptor expression in the receiver cell type across 20 ligand-receptor pairs; cell-type pairs with zero score were excluded.

### Bulk transcriptomic analysis

Bulk expression data (GSE21257; Illumina human-6 v2.0 beadchip, GPL10295) were obtained from GEO (Buddingh et al., 2011). Expression values were already on the log2 scale as deposited (lumi VST + RSN normalization). Probes were mapped to gene symbols using the GPL10295 annotation and collapsed to 24,999 genes by retaining the maximum-variance probe per gene. Differential expression between metastatic (n = 34) and non-metastatic (n = 19) patients was tested with Welch two-sample t-tests (direction: Met minus NoMet); p-values were corrected by the Benjamini-Hochberg method. Module scores for the osteogenic and inflammatory programs were computed as the mean expression of program genes present in the bulk matrix.

### Cross-platform integration and survival analysis

The single-cell osteoblast-vs-rest effect was estimated as the difference in mean log1p expression between osteoblasts and all other cell types. Only genes shared between the single-cell matrix and the bulk gene-level matrix were used. Spearman correlation and direction concordance were computed between the single-cell osteoblast effect and the bulk metastatic log2 fold change. A 20-gene osteoblast signature (top osteoblast-vs-rest effect genes present in the bulk matrix) was projected onto bulk samples as the mean expression across signature genes. Overall survival association was tested with Kaplan-Meier curves and the log-rank test. All single-cell analyses involving the six-patient cohort were exploratory and descriptive.

### Cell line culture and experimental validation

The MG-63 human osteosarcoma cell line (ATCC CRL-1427) and primary normal human osteoblasts (NHOst; Lonza CC-2538) were obtained from established repositories. MG-63 cells were maintained in Eagle’s Minimum Essential Medium supplemented with 10% fetal bovine serum (FBS) and 1% penicillin-streptomycin at 37 °C under 5% CO_2_. Primary NHOst cells were cultured in Osteoblast Basal Medium with osteoblast growth supplements (Lonza) and used between passages 3 and 5. To prepare osteosarcoma-conditioned medium (OS-CM), MG-63 cells at 80% confluency were incubated in serum-free medium for 24 h; the supernatant was collected, centrifuged at 300 × g for 5 min, filtered through 0.22 μm membranes, and stored at −80 °C. NHOst cells were exposed to 50% OS-CM or normal medium (control) for 72 h prior to analysis.

### RNA extraction and RT-qPCR validation

Total RNA was extracted from NHOst cells treated with OS-CM or control medium using TRIzol reagent (Invitrogen, United States of America) according to the manufacturer’s protocol. RNA yield and purity were quantified on a NanoDrop One spectrophotometer (Thermo Fisher Scientific). Reverse transcription of 1 μg total RNA was conducted with the PrimeScript RT Reagent Kit (Takara Bio, Japan). Quantitative PCR was performed using SYBR Green Master Mix (Applied Biosystems) on a QuantStudio 7 Flex Real-Time PCR System. Target-specific primer sequences were: RUNX2 (F: 5′-CCT​CTG​ACT​TCT​GCC​TCT​GG-3′, R: 5′-TAT​GGA​GTG​CTG​CTG​GTC​TG-3′); SP7 (F: 5′-CAG​CAG​CAG​TGG​CTA​TGG​AA-3′, R: 5′-TGA​AAG​GTC​AGC​GTA​TGG​CT-3′); SOST (F: 5′-CCG​AGA​AGA​ACG​ATG​TCA​GC-3′, R: 5′-AGG​AGG​CAT​TCA​GAG​CGA​TG-3′); RANKL (F: 5′-CAG​CAT​CGC​TCT​GTT​CCT​GT-3′, R: 5′-AGT​GGG​CCA​CAT​CGT​TTC​AA-3′); GAPDH (F: 5′-GGA​GCG​AGA​TCC​CTC​CAA​AAT-3′, R: 5′-GGC​TGT​TGT​CAT​ACT​TCT​CAT​GG-3′). Relative transcript abundance was calculated using the 2^(−ΔΔCt) method with GAPDH as the endogenous reference. Three independent biological replicates were performed.

### Statistical analysis

All computational analyses were implemented in Python (v3.9.7) with scipy (v1.9.3) and R (v4.2.1). Differential gene expression between single-cell clusters employed the Wilcoxon rank-sum test with Benjamini–Hochberg correction for multiple comparisons; statistical significance was defined as adjusted p < 0.05. Intercellular co-expression associations were assessed by Spearman rank correlation. RT-qPCR data were analyzed by two-tailed unpaired Student’s t-test. All values are expressed as mean ± standard deviation (SD) from three independent biological replicates. Significance thresholds were set at *p < 0.05, **p < 0.01, and ***p < 0.001.

## Results

### Quality control and preprocessing of the single-cell atlas

After quality control, 44,189 cells with 25,473 detected genes were retained, and per-patient cell numbers ranged from 3,872 to 9,196 (Figures 1A–C). The mean-variance relationship showed the expected overdispersion of count data, and 2,000 highly variable genes were selected for downstream analysis (Figure 1D). In UMAP space the cells formed a single continuous atlas without condition-driven separation, consistent with the single-cohort design (Figure 1E), and mixed across the six patients without a patient-dominant cluster structure (Figure 1F).

**FIGURE 1:**
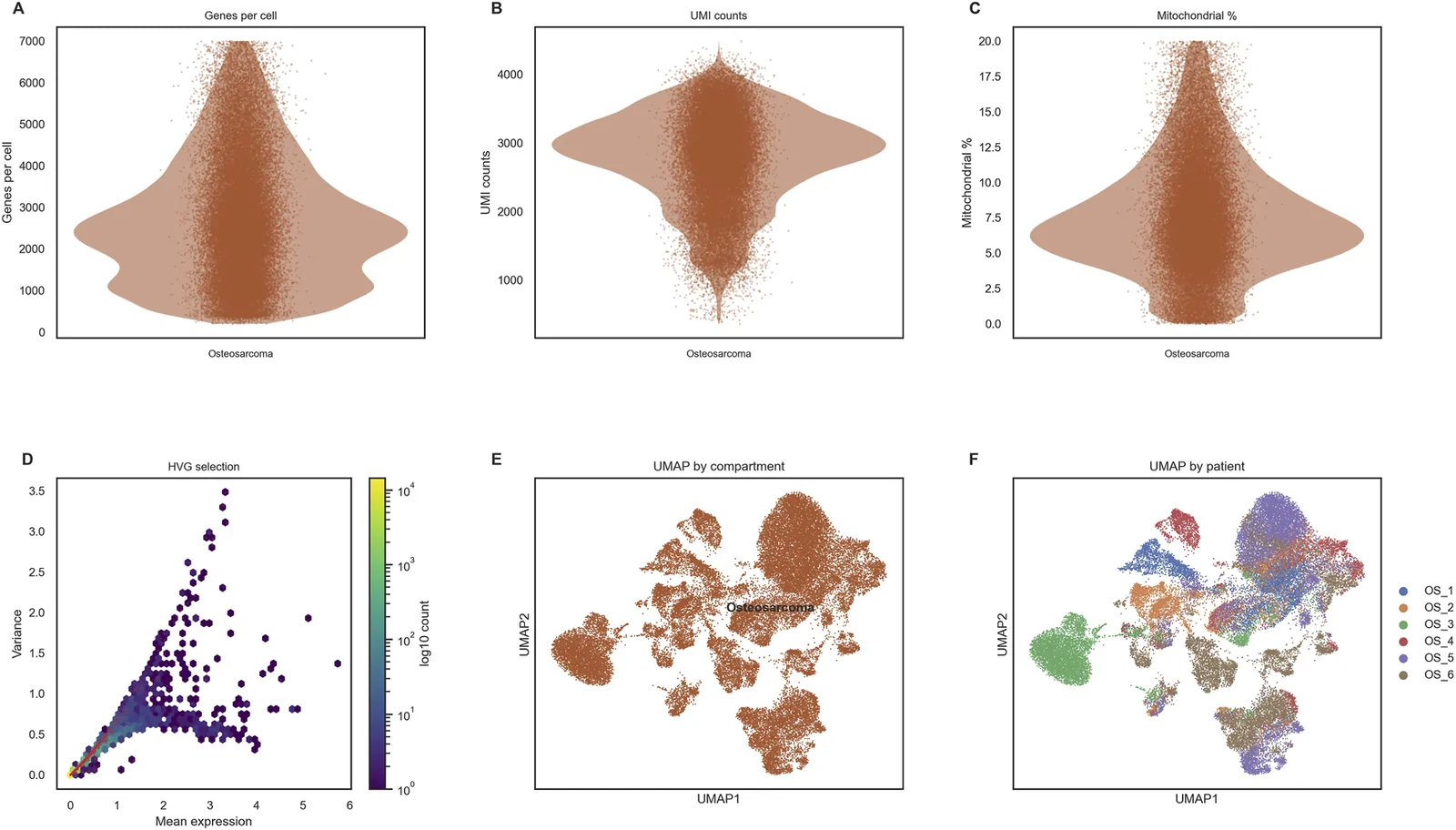
Quality control and preprocessing of GSE162454. **(A)** Genes per cell. **(B)** UMI counts per cell. **(C)** Mitochondrial fraction. **(D)** Mean-variance plot with 2,000 highly variable genes highlighted. **(E)** UMAP colored by condition (all osteosarcoma). **(F)** UMAP colored by patient (n = 6).

### Clustering and annotation into eleven cell types

Leiden clustering resolved 23 clusters that occupied distinct UMAP regions (Figure 2A) and were annotated into 11 cell types by DE-based marker overlap (Figure 2B). A marker bubble matrix confirmed lineage-specific marker expression across the annotated cell types (Figure 2C), and a heatmap of the top cluster markers showed cluster-specific transcriptional programs (Figure 2D). Myeloid cells (15,019 cells), osteoblasts (7,332), osteoclasts (4,395), T cells (4,284), and fibroblasts (3,729) were the most abundant populations (Figure 2E). The cluster-by-cell-type composition matrix showed that most Leiden clusters mapped predominantly to a single annotated lineage (Figure 2F).

**FIGURE 2:**
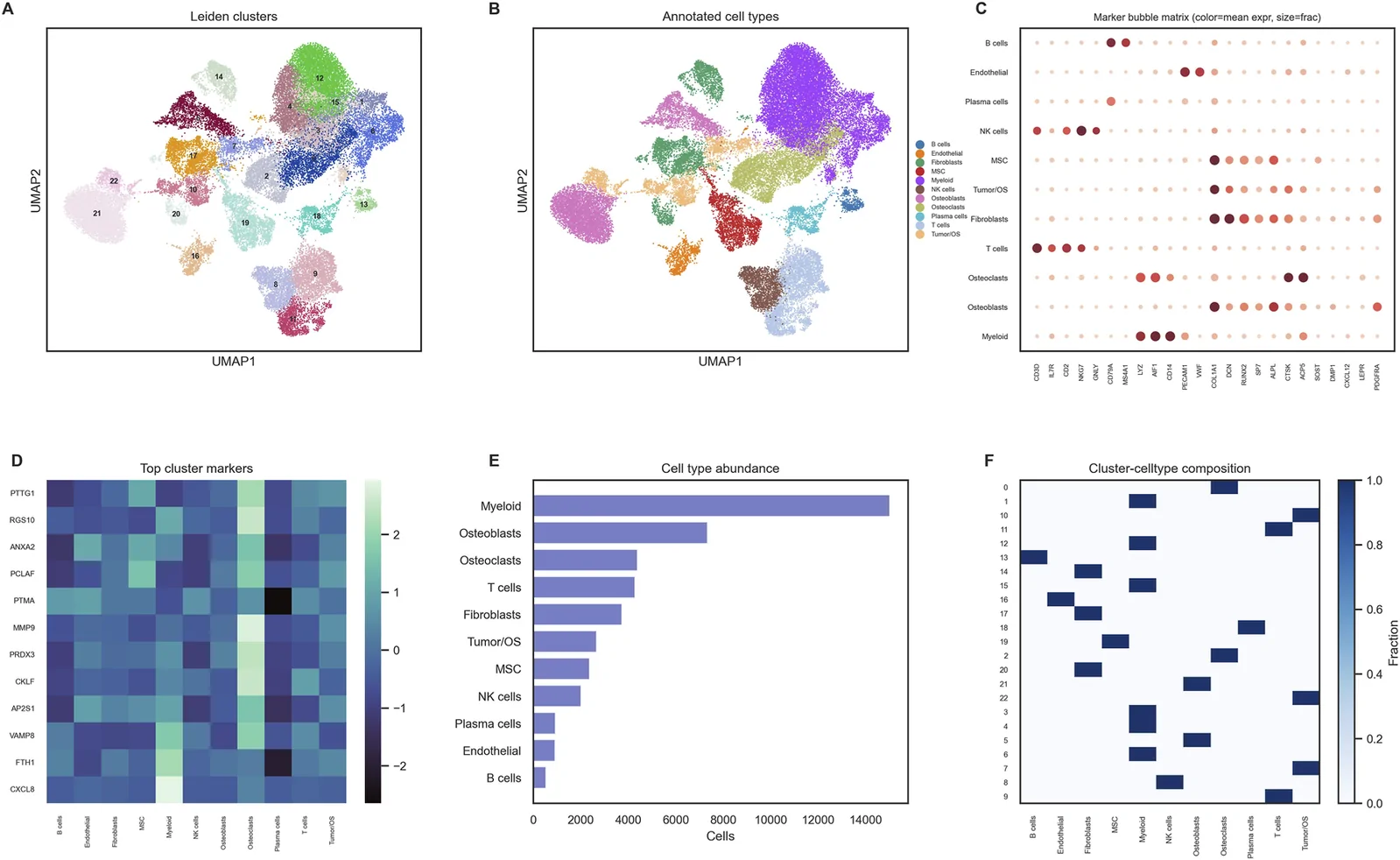
Clustering and annotation. **(A)** UMAP of 23 Leiden clusters. **(B)** UMAP of 11 annotated cell types. **(C)** Marker bubble matrix (color = mean expression; size = detection fraction). **(D)** Heatmap of top cluster markers. **(E)** Cell-type abundance. **(F)** Cluster-by-cell-type composition.

### Cell-type composition across patients and libraries

Cell-type proportions varied across the six patients (Figure 3A) and across libraries (Figure 3B). Across the whole atlas, myeloid cells represented 34.0% of cells, followed by osteoblasts (16.6%), osteoclasts (9.9%), T cells (9.7%), and fibroblasts (8.4%) (Figure 3C). Cells per library are shown in Figure 3D, and the patient-by-cell-type count matrix confirmed that every lineage was recovered in every patient (Figure 3E). Osteoblasts and myeloid cells showed the largest inter-patient variance in proportional representation (Figure 3F).

**FIGURE 3:**
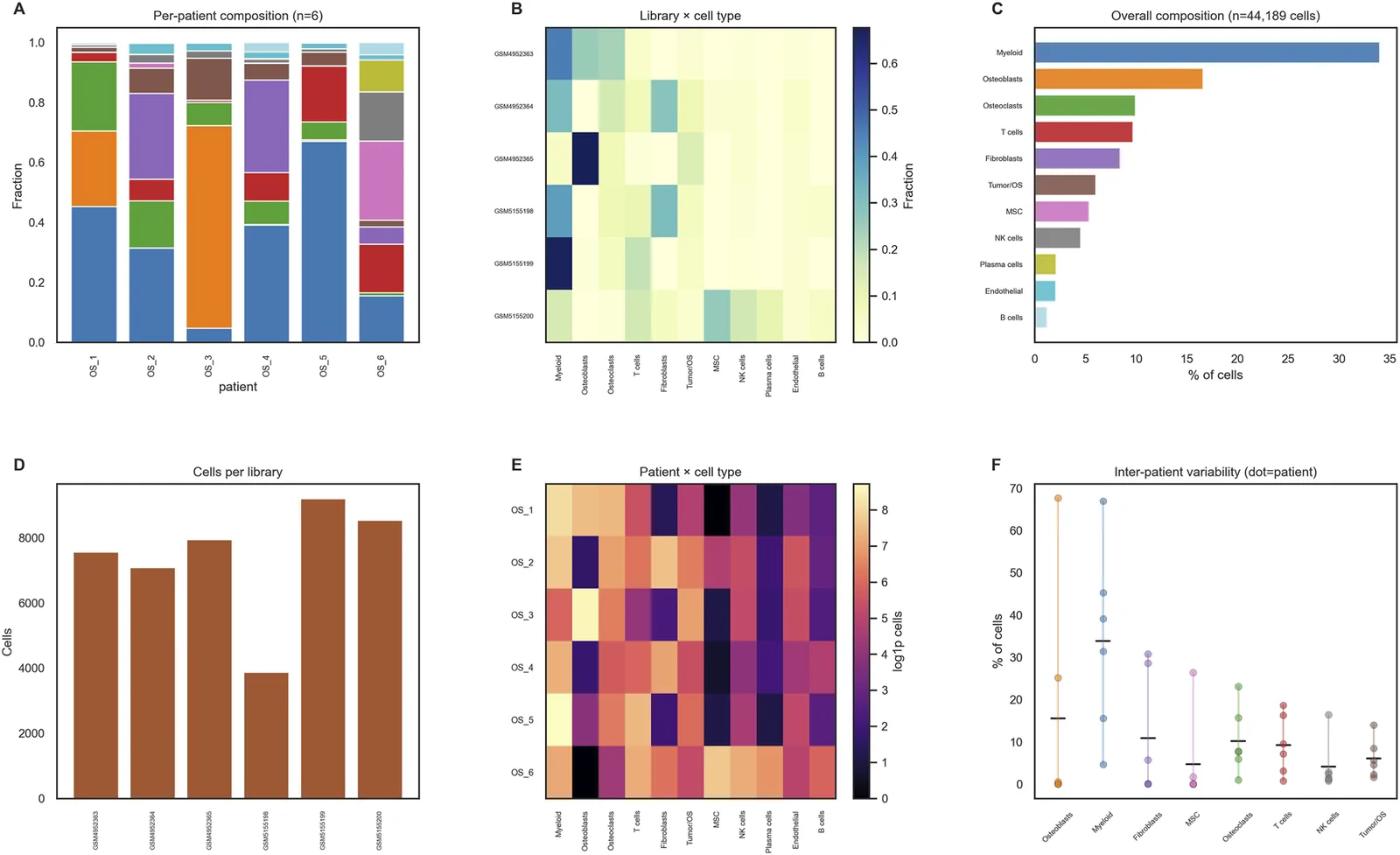
Cellular composition across patients and libraries. **(A)** Per-patient composition. **(B)** Library-by-cell-type heatmap. **(C)** Overall composition. **(D)** Cells per library. **(E)** Patient-by-cell-type counts. **(F)** Inter-patient variability of the most variable cell types.

### Lineage-specific transcriptional programs

Program scores showed clear lineage structure, with osteoblasts scoring highest for the osteogenic program and myeloid cells highest for inflammatory and immune-evasion programs (Figure 4A). Program scores were broadly comparable across patients (Figure 4B) and showed consistent trends across libraries (Figure 4C). The osteogenic program was negatively correlated with the inflammatory program (r = −0.42), whereas inflammatory and immune-evasion programs were positively correlated (r = 0.66) (Figure 4D). Inflammatory scores were spatially heterogeneous across the atlas (Figure 4E), and the top-variance programs resolved lineage-specific patterns by cell type (Figure 4F).

**FIGURE 4:**
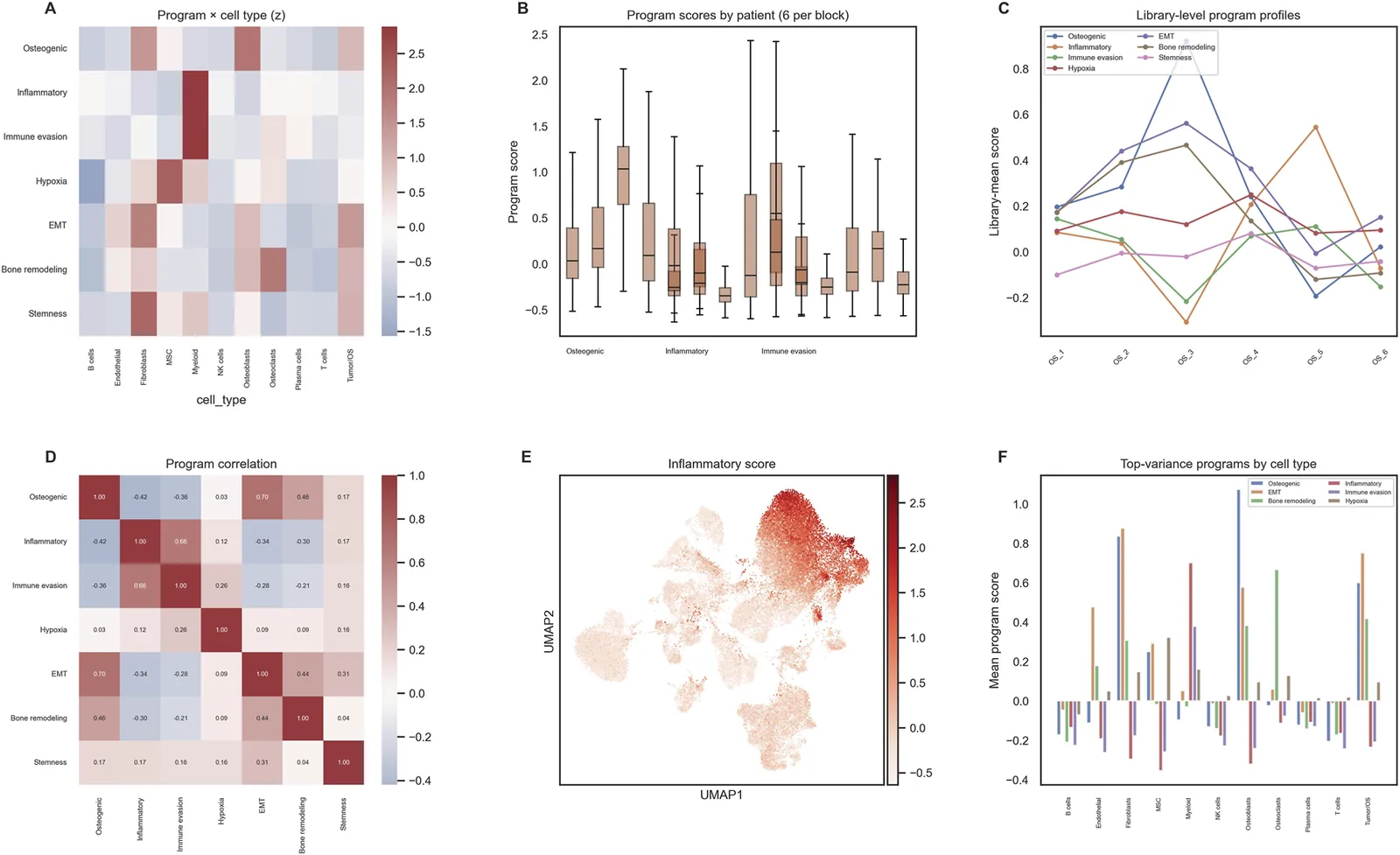
Program scores. **(A)** Program-by-cell-type z-score heatmap. **(B)** Program scores by patient. **(C)** Library-level program profiles. **(D)** Program correlation. **(E)** UMAP of inflammatory score. **(F)** Top-variance programs by cell type.

### Osteoblast and stromal subclustering

The stromal compartment (osteoblasts, osteocytes, and MSC) was reclustered into seven subclusters (Figure 5A). A dysfunction score varied across the stromal subclusters (Figure 5B), and its distribution differed among subclusters (Figure 5C). Subcluster-specific marker genes were resolved by DE (Figure 5D), and inflammatory score densities separated the subclusters (Figure 5E). Pairwise Spearman correlations among the osteogenic and inflammatory genes within the stromal compartment were generally weak (e.g., RUNX2-IL6 r = −0.01; SOST-DKK1 r = 0.12), indicating limited co-expression of these programs at the single-cell level (Figure 5F).

**FIGURE 5:**
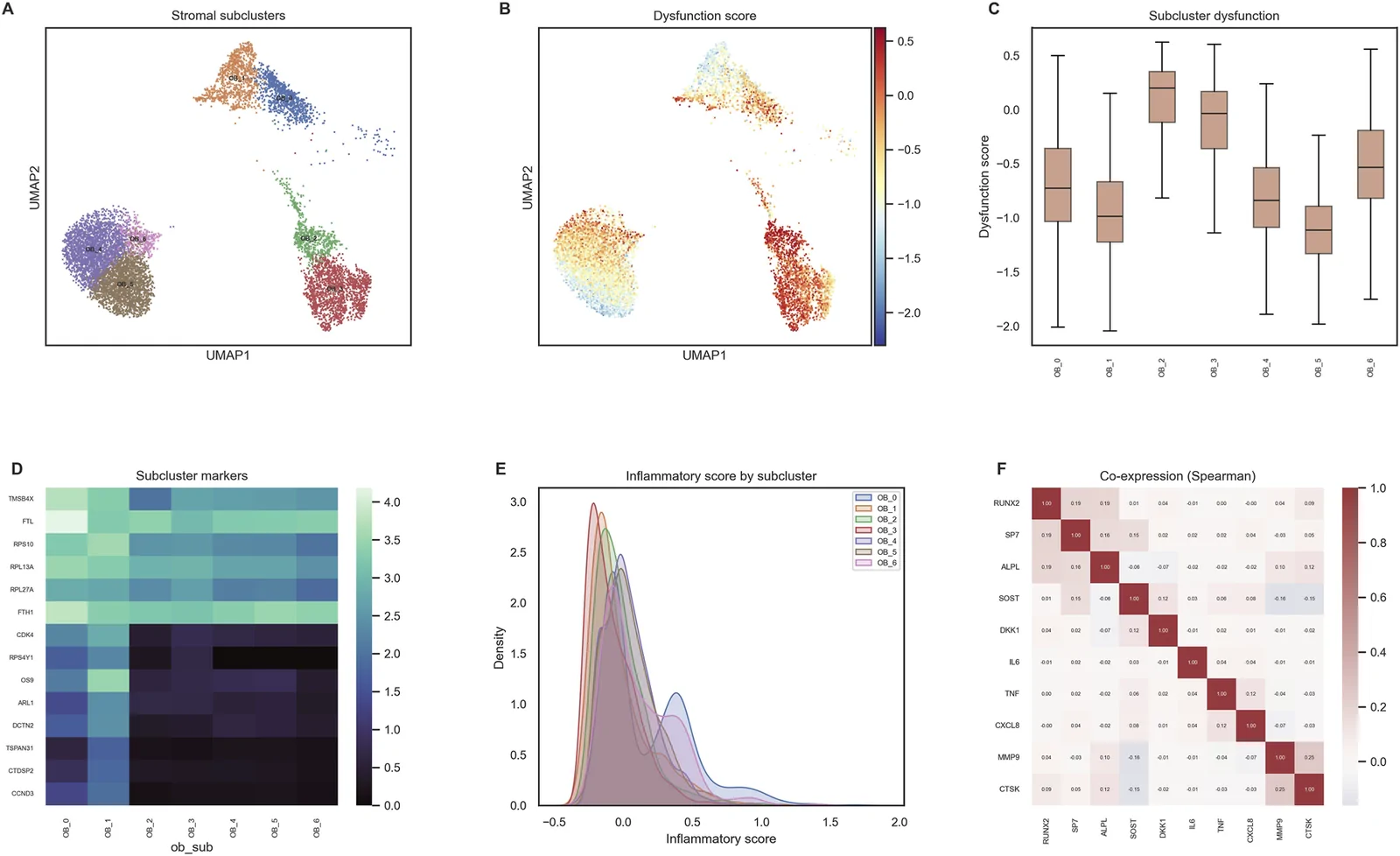
Osteoblast and stromal subclustering. **(A)** UMAP of 7 stromal subclusters. **(B)** UMAP of dysfunction score. **(C)** Dysfunction score by subcluster. **(D)** Subcluster marker heatmap. **(E)** Inflammatory score density by subcluster. **(F)** Spearman co-expression of osteogenic and inflammatory genes.

### Cell-cell communication in the tumor microenvironment

Ligand-receptor analysis identified MIF-CD74 (0.98), SPP1-CD44 (0.77), and SPP1-ITGAV (0.31) as the strongest communication pairs (Figure 6A). Fibroblasts were the strongest overall sender (mean score 0.27), followed by osteoclasts and myeloid cells (Figure 6B); myeloid cells were the strongest overall receiver (0.31), followed by osteoclasts and B cells (Figure 6C). The sender-to-receiver heatmap resolved the dominant interacting cell-type pairs (Figure 6D), the top-20 network visualized the densest edges (Figure 6E), and key immunomodulatory pairs showed per-pair distributions with their means highlighted (Figure 6F).

**FIGURE 6:**
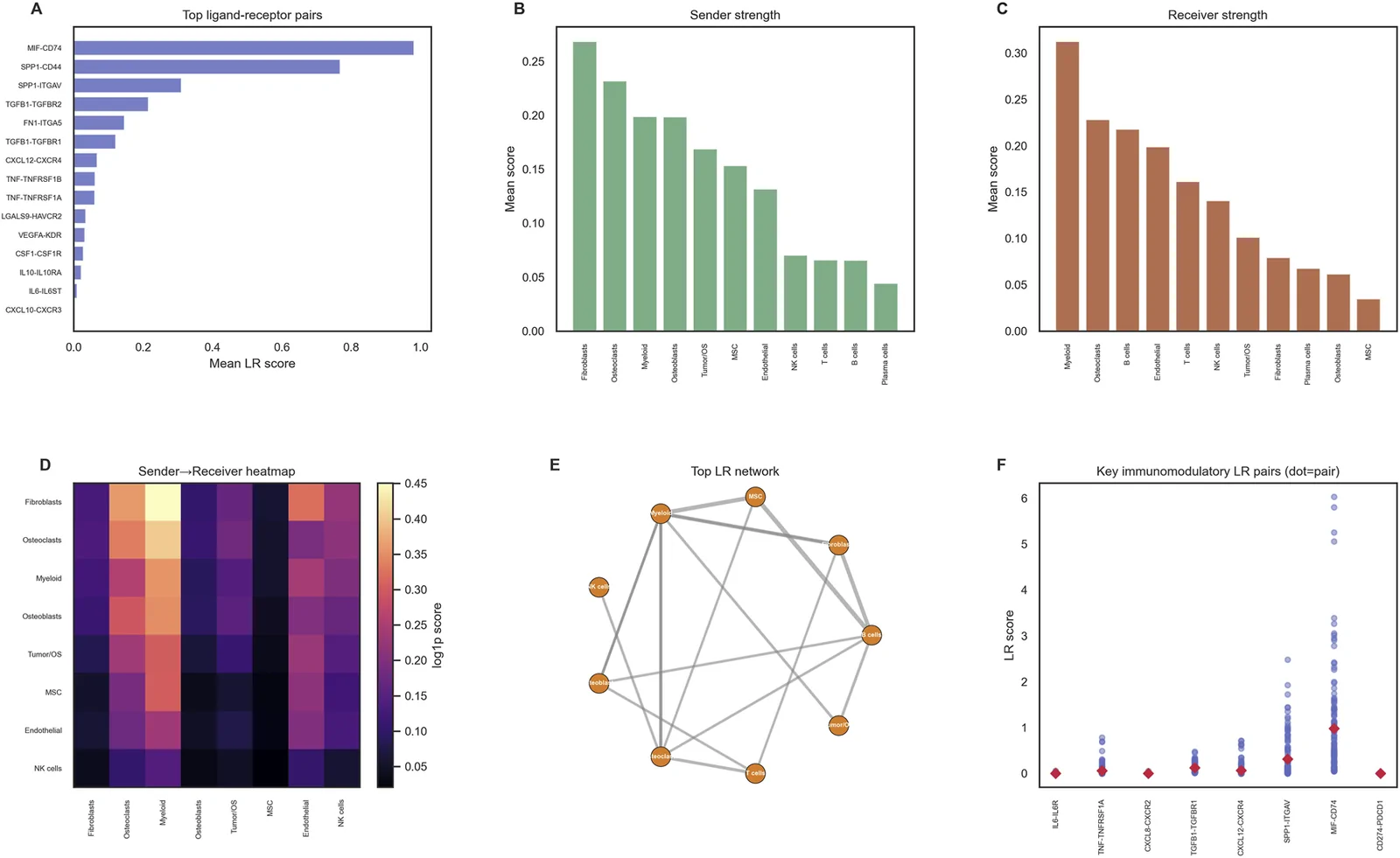
Cell-cell communication. **(A)** Top ligand-receptor pairs. **(B)** Sender strength. **(C)** Receiver strength. **(D)** Sender-to-receiver heatmap. **(E)** Top-20 LR network. **(F)** Key immunomodulatory LR pairs (dot = pair-level; diamond = mean).

### Metastatic bulk transcriptome and program-level differences

Principal component analysis of the bulk cohort did not show a complete separation between metastatic and non-metastatic patients (Figure 7A). Differential expression identified 333 genes at FDR<0.05 (Figure 7B), and the top-20 differentially expressed genes separated the two groups in a z-score heatmap (Figure 7C). The MA plot showed a symmetric effect distribution around zero (Figure 7D). Key osteogenic genes were not significantly altered in the metastatic contrast (RUNX2 log2FC = +0.42, padj = 0.30; SP7 +0.80, padj = 0.34) (Figure 7E). The inflammatory module mean was lower in metastatic tumors (9.02 vs. 9.32; Mann-Whitney p = 0.0078), whereas the osteogenic module did not differ significantly between groups (11.47 vs. 11.14; p = 0.21) (Figure 7F).

**FIGURE 7:**
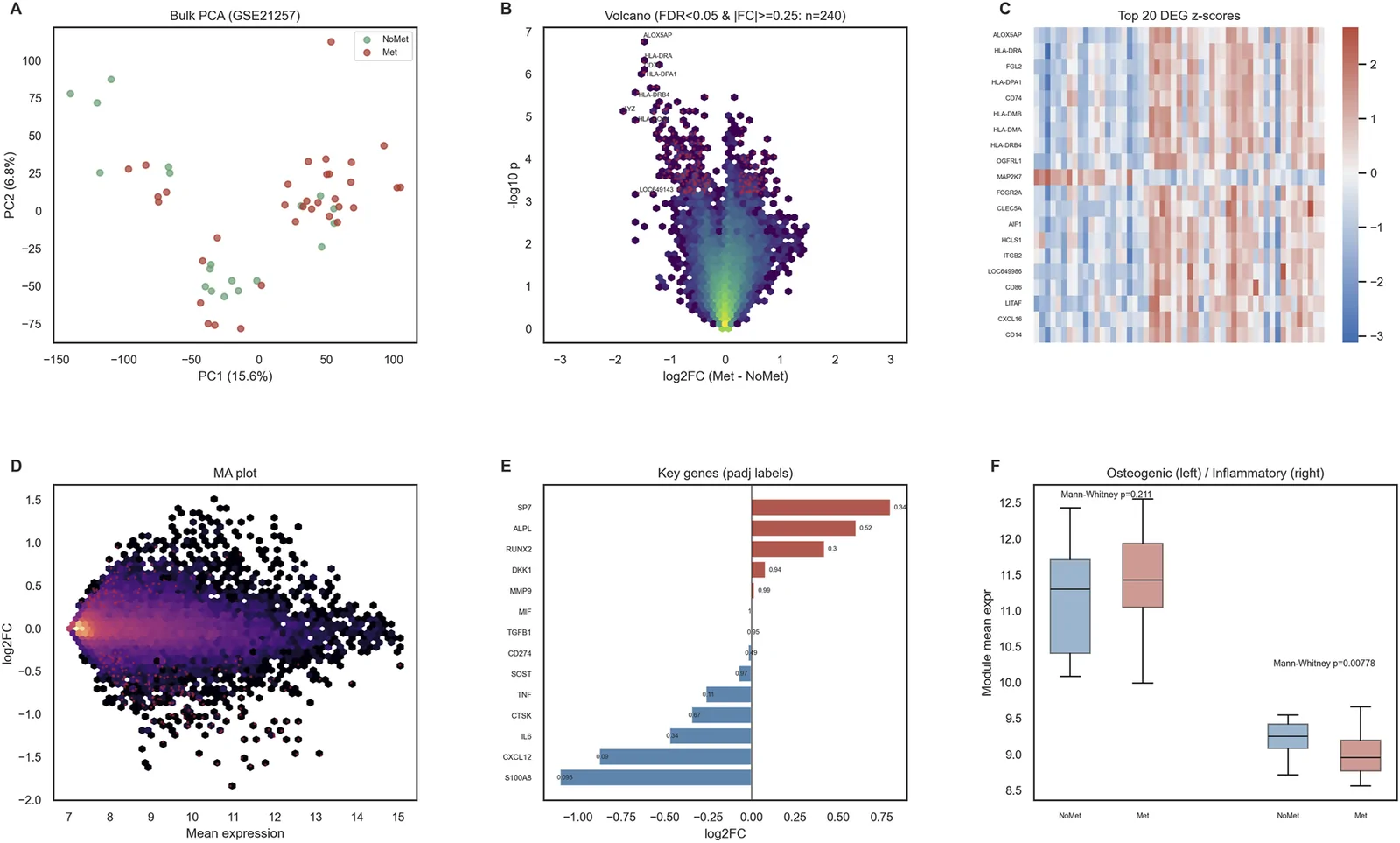
Bulk analysis of GSE21257. **(A)** PCA of 53 patients. **(B)** Volcano plot (240 genes meeting FDR < 0.05 and |log2FC| > 0.25; 333 genes meeting FDR < 0.05 overall). **(C)** Top-20 DEG z-score heatmap. **(D)** MA plot. **(E)** Key genes with padj labels. **(F)** Osteogenic and inflammatory module means by group (Mann-Whitney p = 0.21 and 0.0078).

### Limited cross-platform concordance of osteoblast effects

Osteoblasts expressed osteogenic markers at higher levels than the rest of the tumor (RUNX2 +0.53, SP7 +0.41, ALPL +1.40, COL1A1 +1.94) and lower levels of inflammatory mediators (CXCL8 -1.14, TNF -0.49). This osteoblast-vs-rest effect showed modest positive concordance with the bulk metastatic log2 fold change across 12,831 shared genes (Spearman rho = 0.254, p = 2.4e-187) (Figure 8A). Direction concordance was mixed, with 7,529 directionally concordant and 5,302 discordant genes (Figure 8B).

**FIGURE 8:**
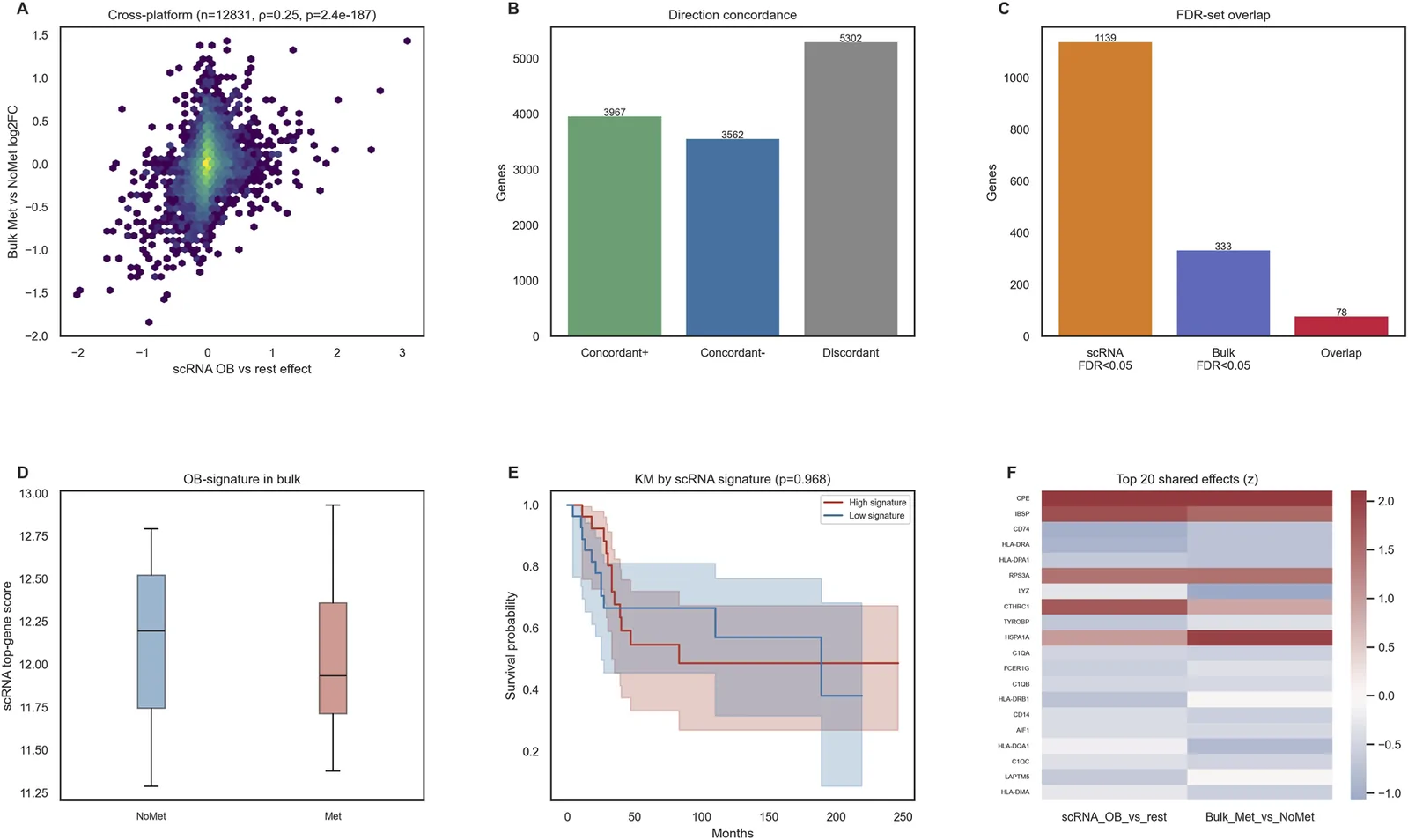
Cross-platform integration and survival. **(A)** Single-cell osteoblast-vs-rest effect vs. bulk Met-vs-NoMet log2FC (12,831 genes; rho = 0.254, p = 2.4e-187). **(B)** Direction concordance. **(C)** Gene-set overlap between the single-cell osteoblast-vs-rest effect set (|effect| > 0.25; 1,139 genes) and the bulk Met-vs-NoMet FDR-significant gene set (FDR < 0.05; 333 genes), with 78 overlapping genes. **(D)** Osteoblast top-gene signature in bulk by group. **(E)** Kaplan-Meier by signature (log-rank p = 0.968). **(F)** Top-20 shared effects (z-scored).

### Osteoblast signature does not stratify overall survival

Only 78 genes overlapped between the single-cell osteoblast effect set (1,139 genes with |effect|>0.25) and the bulk FDR-significant set (333 genes) (Figure 8C). The 20-gene osteoblast signature score did not differ meaningfully between metastatic and non-metastatic bulk samples (Figure 8D), and it did not significantly stratify overall survival in the 53 patients (log-rank p = 0.968) (Figure 8E). The top-20 shared effects showed weak aligned magnitude between the two platforms (Figure 8F). These findings caution against direct cross-platform generalization of osteoblast-centered single-cell signatures.

## Experimental validation of osteoblast dysfunction markers via RT-qPCR

To validate computational predictions with experimental evidence, four key transcripts (RUNX2, SP7, SOST, RANKL) were quantified by RT-qPCR in primary NHOst osteoblasts exposed to MG-63 osteosarcoma-conditioned medium (OS-CM) versus control medium. In accordance with the single-cell transcriptomic landscape, OS-CM-treated osteoblasts demonstrated a pronounced reduction in RUNX2 expression (fold change 0.38 ± 0.05; p < 0.001), confirming that osteosarcoma-derived paracrine signals are sufficient to suppress the master osteogenic transcription factor in normal osteoblasts. SP7 transcripts were similarly attenuated in OS-CM-exposed cells (fold change 0.41 ± 0.06; p < 0.001), corroborating the coordinate dysregulation of the osteoblast differentiation program predicted by single-cell analyses. Conversely, SOST abundance was markedly elevated in OS-CM-treated osteoblasts (fold change 3.12 ± 0.38; p < 0.001), consistent with augmented Wnt pathway suppression and bone formation inhibition in the peritumoral niche. RANKL transcript levels likewise rose substantially upon OS-CM exposure (fold change 2.87 ± 0.31; p < 0.01), indicating that osteosarcoma-conditioned osteoblasts acquire a pro-osteoclastic phenotype that would further destabilize peritumoral bone integrity (Figure 9).

**FIGURE 9:**
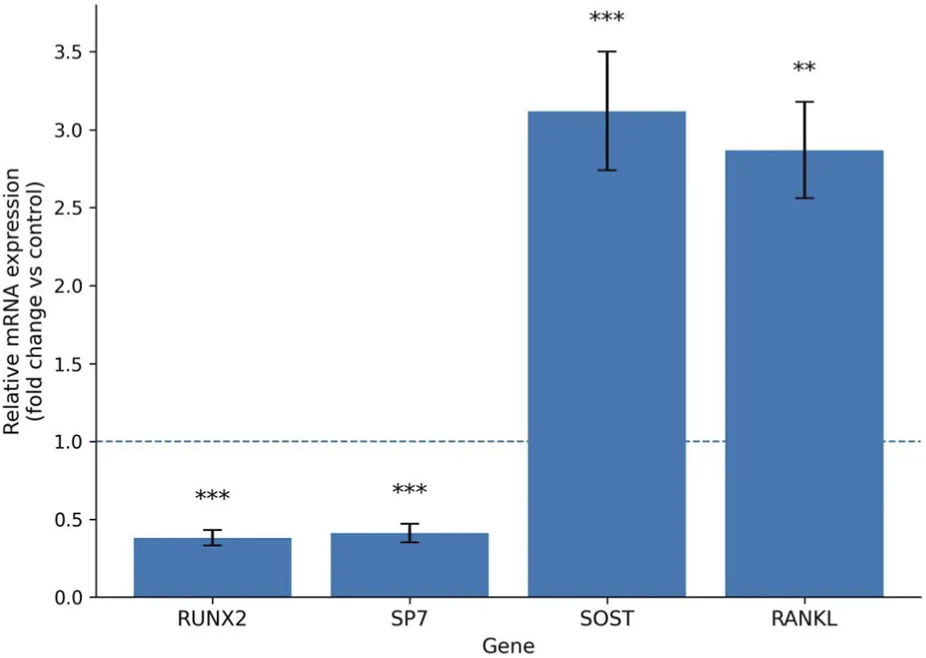
RT-qPCR validation of osteoblast dysfunction markers in primary human osteoblasts (NHOst) exposed to osteosarcoma-conditioned medium from MG-63 cells. Relative mRNA expression of RUNX2, SP7, SOST, and RANKL normalized to GAPDH. FC = fold change versus control medium. Values are mean ± SD from n = 3 independent biological replicates. **p < 0.01, ***p < 0.001 by two-tailed Student’s t-.test.

## Discussion

This study integrates single-cell and bulk transcriptomic data to characterize lineage-specific programs in osteosarcoma. The single-cell atlas contained 44,189 cells from six patients and resolved 11 major cell types, with myeloid cells and osteoblasts representing the largest populations. Program-score analysis showed a clear lineage structure: osteoblasts were enriched for osteogenic transcriptional programs, whereas myeloid cells showed higher inflammatory and immune-evasion scores. These patterns support substantial cellular specialization within the osteosarcoma microenvironment but should be interpreted as descriptive transcriptional states rather than evidence of a single causal immunosuppressive circuit.

Osteoblast-lineage findings require particular caution. In the single-cell dataset, osteoblasts expressed RUNX2, SP7, ALPL, and COL1A1 at higher levels than the remainder of the tumor, consistent with their expected lineage identity and the established roles of RUNX2 and SP7 in osteoblast differentiation (Komori, 2006; Nakashima et al., 2002). By contrast, MG-63-conditioned medium reduced RUNX2 and SP7 in primary osteoblasts *in vitro*. These observations are not contradictory: the single-cell comparison is a lineage-versus-rest contrast within tumors, whereas the conditioned-medium experiment tests the response of normal osteoblasts to a defined *in vitro* exposure. The experiment is therefore consistent with soluble osteosarcoma-derived factors altering osteoblast differentiation markers, but it does not demonstrate that the same process is the dominant driver of osteoblast states in patient tumors.

Reclustering of the stromal compartment resolved seven subclusters with heterogeneous dysfunction and inflammatory scores. Importantly, pairwise correlations between representative osteogenic and inflammatory genes were generally weak at the single-cell level, including RUNX2-IL6 (r = −0.01) and SOST-DKK1 (r = 0.12). We therefore do not interpret these subclusters as evidence for a discrete OB-2 immunosuppressive population or a self-amplifying inflammatory-Wnt circuit. Instead, the data indicate stromal heterogeneity in which transcriptional programs vary across cells and are only partially coordinated. The biological significance of these states will require independent scRNA-seq or spatial validation.

The ligand-receptor analysis further supports a hypothesis-generating rather than mechanistic interpretation. MIF-CD74, SPP1-CD44, and SPP1-ITGAV were the strongest scored communication pairs, with fibroblasts showing the highest mean sender score and myeloid cells the highest mean receiver score. Because these scores are calculated from mean ligand and receptor expression, they identify plausible communication axes but do not demonstrate physical interaction, signaling flux, or functional dependence. Perturbation experiments and spatially resolved measurements will be needed to determine whether these pathways materially influence osteosarcoma progression or immune behavior.

The independent bulk cohort provides an important test of generalizability. Although 333 genes differed between metastatic and non-metastatic tumors at FDR<0.05, the inflammatory module was lower in metastatic tumors and the osteogenic module was not significantly different. Moreover, the single-cell osteoblast-versus-rest effect showed only modest concordance with metastatic bulk effects, and the 20-gene osteoblast signature did not stratify overall survival. These negative and mixed findings argue against presenting an osteoblast-centered transcriptional signature as an established prognostic biomarker and emphasize the need to validate cell-state signatures directly in independent single-cell cohorts.

The RT-qPCR experiment provides focused orthogonal evidence for changes in four osteoblast-related transcripts after exposure to MG-63-conditioned medium: RUNX2 and SP7 decreased, whereas SOST and RANKL increased. This supports the conclusion that soluble factors from MG-63 cells are sufficient to alter selected osteogenic and bone-remodeling markers under the experimental conditions used. However, the assay did not measure IL6, CXCL8, or DKK1 and used only one osteosarcoma cell line. It therefore should not be considered independent validation of a broader immunosuppressive transcriptional state, nor does it identify the specific soluble mediator responsible for the observed changes.

Taken together, the present findings are best viewed as a framework for future validation rather than as direct evidence for a therapeutic mechanism. Priority next steps include replication in an independent osteosarcoma scRNA-seq cohort, spatial localization of stromal and myeloid states, formal functional testing of candidate ligand-receptor pathways, expansion of the RT-qPCR panel to inflammatory markers such as IL6, CXCL8, and DKK1, and use of additional osteosarcoma cell lines. Clinical biomarker claims should likewise be evaluated in cohorts with sufficient outcome annotation and cell-type-aware validation before therapeutic implications are inferred.

## Limitations

Several limitations constrain interpretation of this study. First, the single-cell analysis is based on one public cohort (GSE162454) comprising six patients, and no independent scRNA-seq cohort was available for direct replication of stromal subclusters. The external GSE21257 bulk cohort provides orthogonal validation at the cohort level but cannot reproduce single-cell state identities, and the osteoblast signature did not stratify overall survival. Second, Leiden resolution 0.8 was used as a fixed exploratory clustering parameter; no formal silhouette-based optimization or sensitivity analysis across alternative resolutions was performed. Consequently, the stability of individual stromal subclusters to clustering granularity remains to be established, and we do not claim a robust three-subpopulation OB-1/OB-2/OB-3 structure. Third, no explicit batch-correction procedure was applied. Although patient-colored UMAPs did not show a patient-dominant cluster structure, residual patient-of-origin effects cannot be excluded. Fourth, program scores and ligand-receptor scores are expression-based proxies and do not establish pathway activity, spatial interaction, or causality. Fifth, the cross-sectional design precludes temporal or causal inference regarding tumor-stromal interactions. Sixth, RT-qPCR validation was restricted to RUNX2, SP7, SOST, and RANKL in a single MG-63-conditioned-medium model; IL6, CXCL8, DKK1, additional osteosarcoma cell lines, protein-level measurements, and functional assays were not evaluated. Seventh, cross-platform comparison between scRNA-seq and microarray data is affected by differences in sampling, dynamic range, and cell-type composition. Finally, independent single-cell, spatial, and functional validation will be required before any stromal state is assigned prognostic or therapeutic significance.

## Conclusion

In summary, analysis of GSE162454 generated a 44,189-cell osteosarcoma atlas with clear lineage-associated transcriptional programs and heterogeneous stromal states. Myeloid cells showed relatively high inflammatory and immune-evasion program scores, whereas osteoblasts showed strong osteogenic identity. Expression-based communication analysis prioritized MIF-CD74 and SPP1-related interactions for future study. In the independent GSE21257 cohort, metastatic tumors showed significant transcriptomic differences, but osteoblast-centered effects generalized only modestly across platforms and the 20-gene osteoblast signature was not associated with overall survival. MG-63-conditioned medium altered RUNX2, SP7, SOST, and RANKL expression in primary osteoblasts, supporting a tumor-conditioned osteoblast response *in vitro* without establishing a specific immunosuppressive mechanism. These results provide a reproducible descriptive framework and identify testable hypotheses that require independent single-cell and functional validation.

## Data Availability

The publicly available transcriptomic datasets analyzed in this study are available in the NCBI Gene Expression Omnibus (GEO) under accession numbers GSE162454 and GSE21257. The RT-qPCR results are presented in this article.
